# Supplementary material for: Structural basis of outer membrane biogenesis by the TamAB translocase
Source: Nat Commun. 2026 Jan 13;17:437. doi: 10.1038/s41467-025-67115-7 (PMC12800296; doi:10.1038/s41467-025-67115-7)
Supplement: Supplementary file 1 — Supplementary Information [file 41467_2025_67115_MOESM1_ESM.pdf]

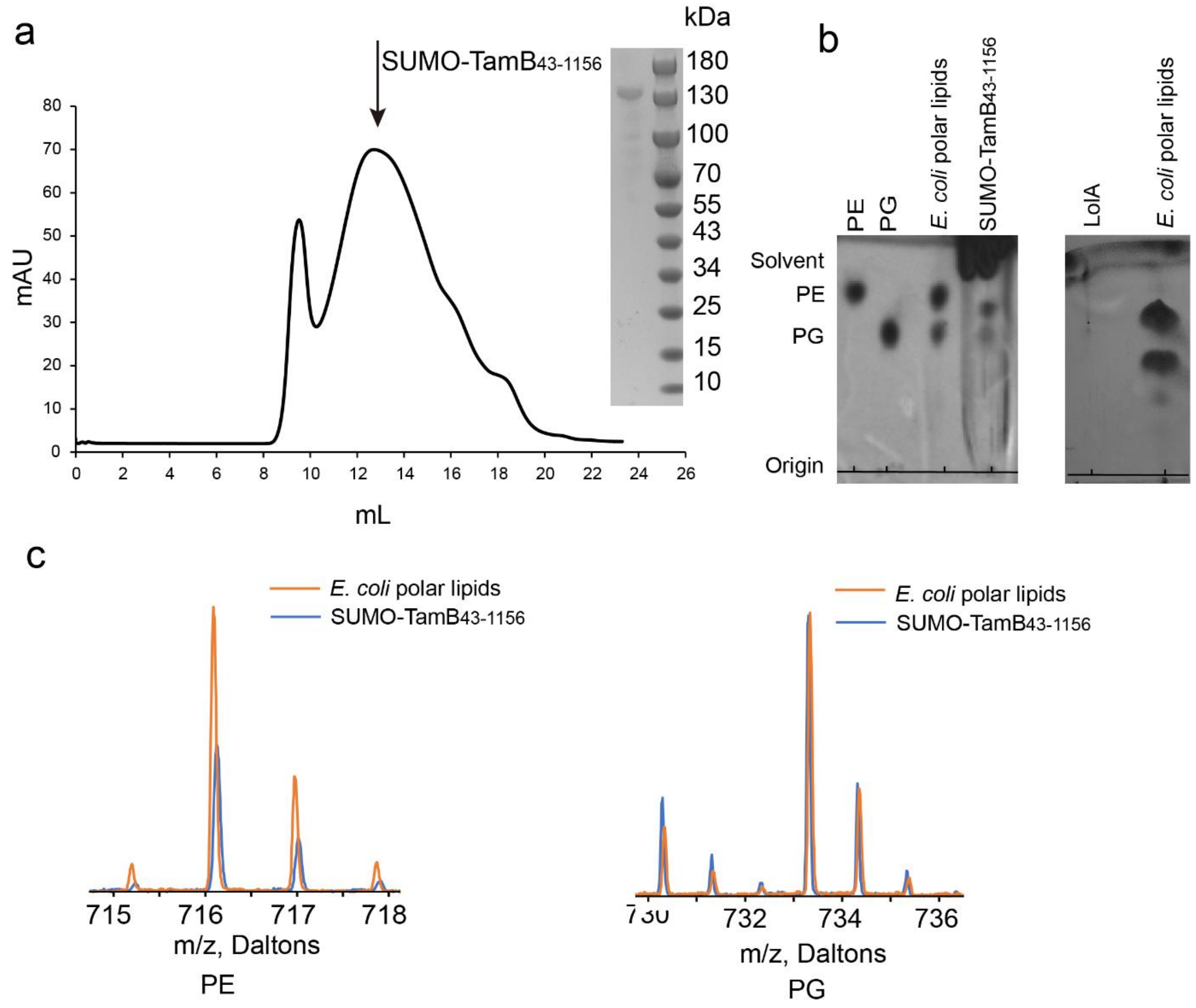

Supplementary Figure. 1. | TamB periplasmic domain binds phospholipid. a, TamB periplasmic domain purification by size-exclusion chromatography. **b,** Phospholipids were extracted from the purified TamB periplasmic domain and detected by thin-layer chromatography. The PE, PG and *E. coli* polar lipids were used as lipid standards. **c,** Phospholipids were extracted from the purified TamB periplasmic domain and detected by 5800 MALDITOF (AB SCIEX, USA) mass spectrometer. The *E. coli* polar lipids were used as lipid standards.

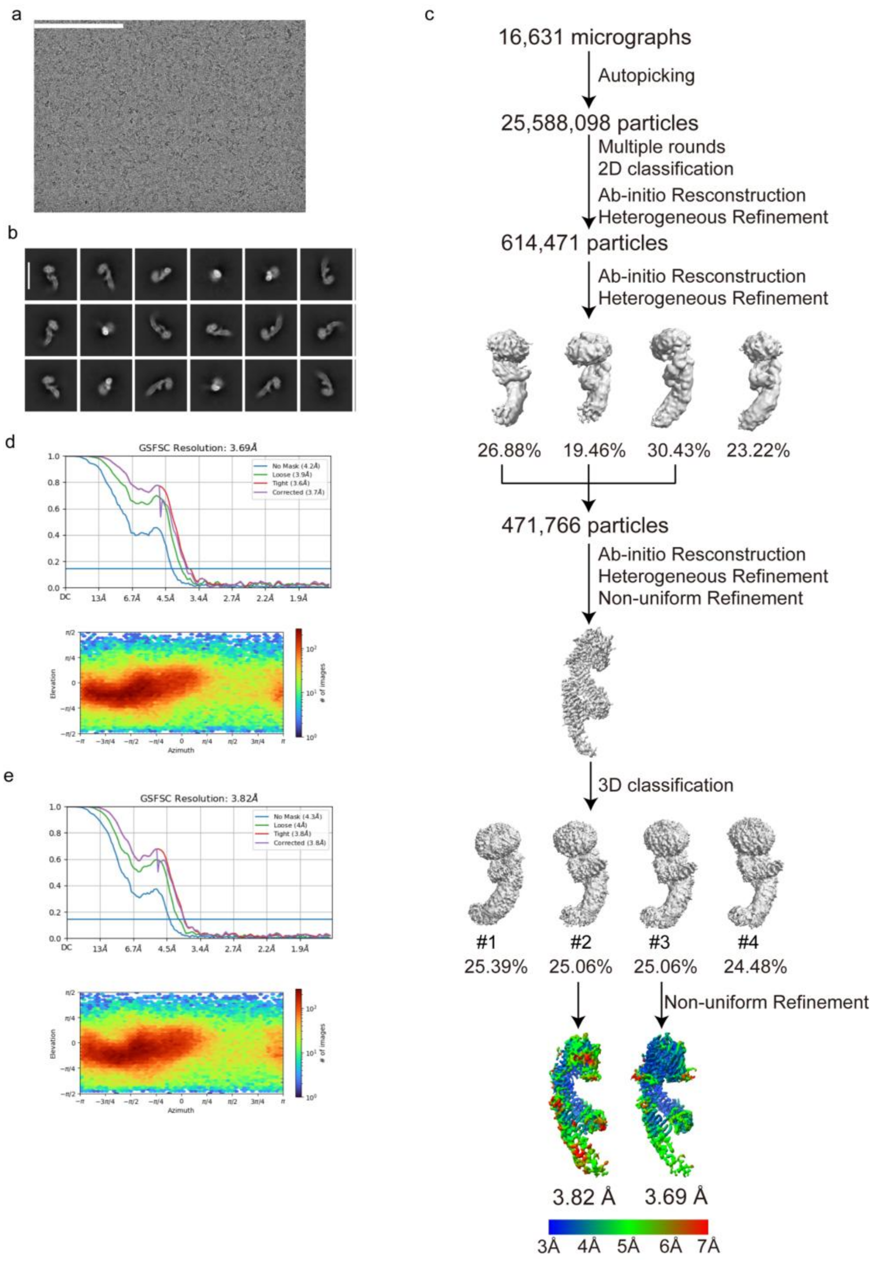

Supplementary Figure. 2. | Flow charts of cryo-EM structure determination of TamAB in two different states. a, A cryo-EM micrograph of single particles of TamAB. The scale bar represents 160 nm. **b,** 2D classes of TamAB. The scale bar represents 140 Å**. c,** Data processing chart. **d,** Gold standard Fourier shell correlation curve of the structure of the hybrid barrel TamAB (upper). Angular distribution of particle projections of the hybrid barrel TamAB. **e,** Gold standard Fourier shell correlation curve of the structure of the non-hybrid barrel TamAB (upper). Angular distribution of particle projections of the non-hybrid barrel TamAB.

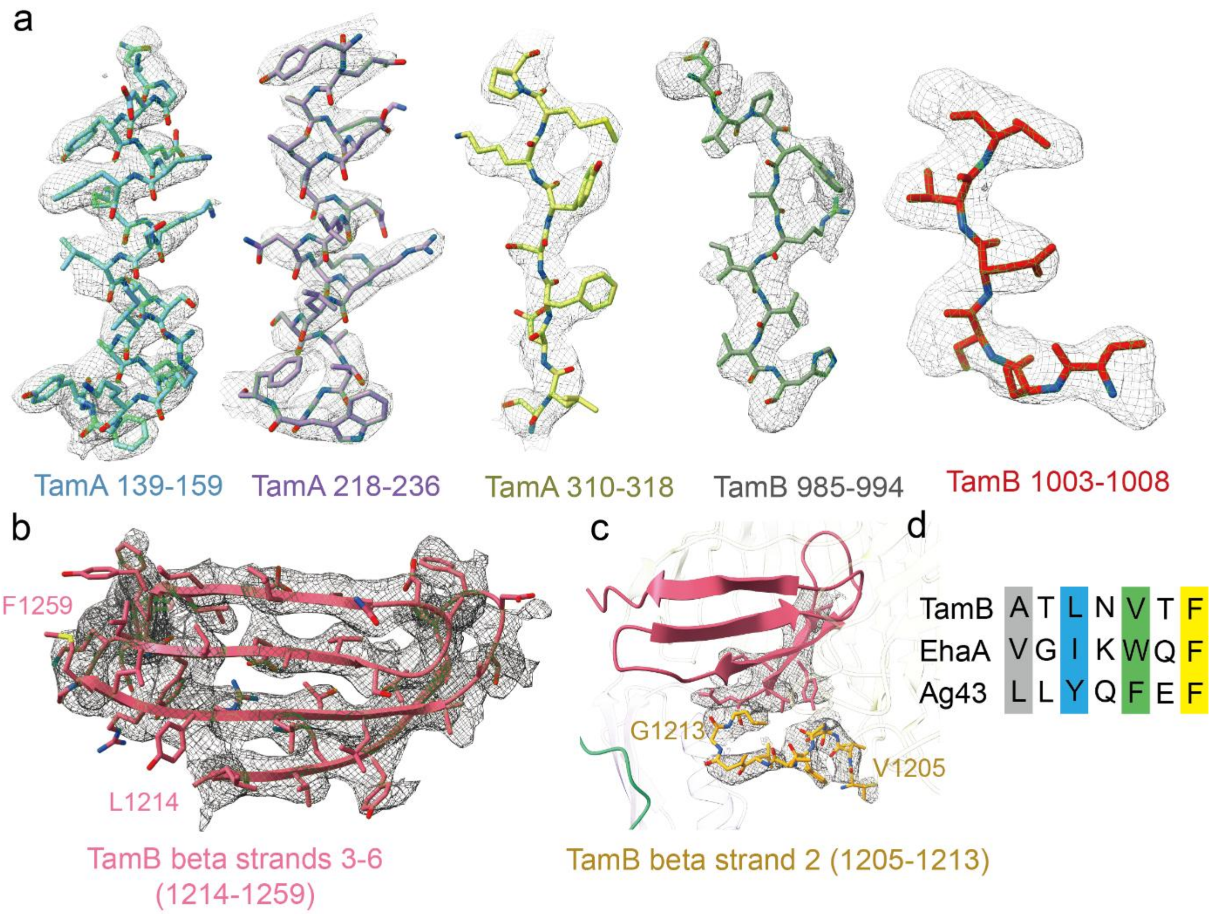

Supplementary Figure. 3. | Cryo-EM densities of TamAB. a, Typical densities of TamA and TamB residues. **b,** Density of the TamB C-terminal β3-β6 in hybrid barrel structure. **c,** The weak density of TamB C-terminal β2 in hybrid barrel structure. **d,** Amino acid sequence alignment of TamB, EhaA and Ag43 beta-signal like sequence.

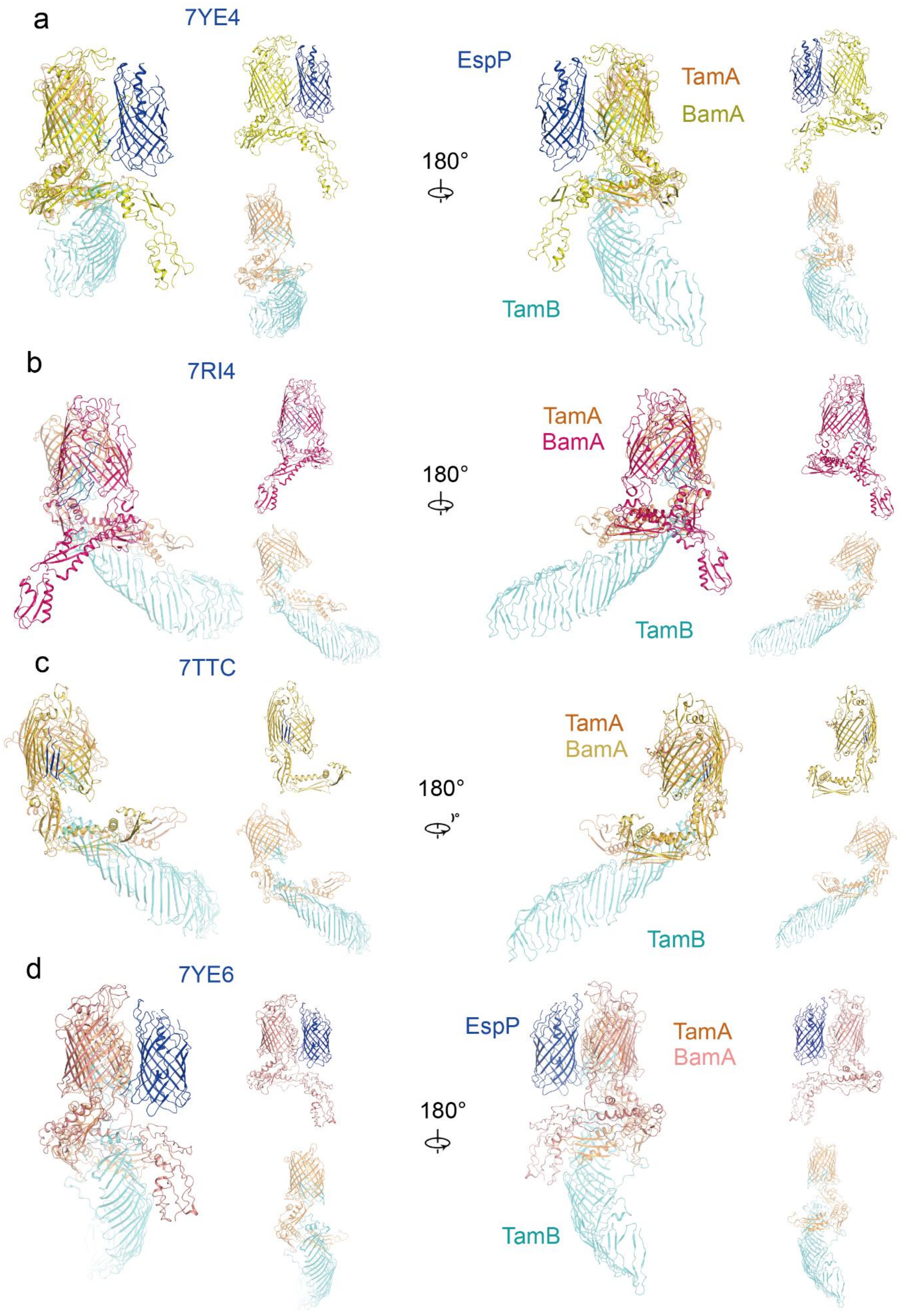
a
7YE4
EspP
TamA
BamA
180°
TamB
b
7RI4
TamA
BamA
180°
TamB
c
7TTC
TamA
BamA
180°
TamB
d
7YE6
EspP
TamA
BamA
180°
TamB

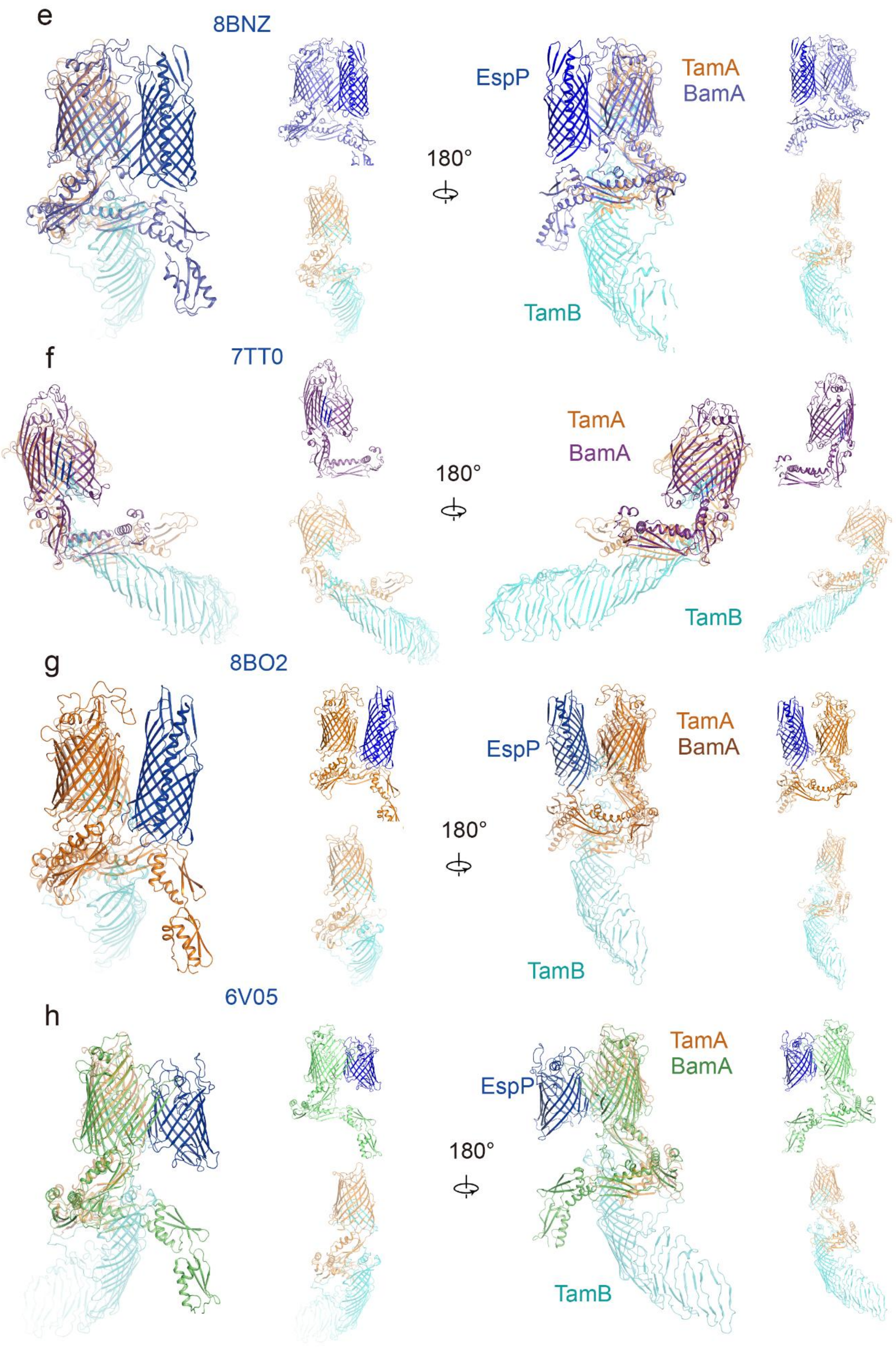
e
8BNZ
EspP
TamA
BamA
180°
TamB
f
7TT0
TamA
BamA
180°
TamB
g
8BO2
EspP
TamA
BamA
180°
TamB
h
6V05
TamA
BamA
EspP
180°
TamB

Supplementary Figure. 4. | Comparison of TamAB hybrid barrel structure with BAM and substrate complexes. a, TamAB hybrid barrel structure superimposition with BAM-EspP complex (PDB code: 7YE4) and with BamA G431C-G781C/EspP N1293C-A1043C mutations. All twelve EspP beta-strands are outside of the BamA barrel. **b,** TamAB hybrid barrel structure superimposition with BAM-EspP complex (PDB code: 7RI4) with four EspP beta-strands outside of the BamA barrel. **c,** TamAB hybrid barrel structure superimposition with BAM-EspP complex (PDB code: 7TTC) with four EspP beta-strands outside of the BamA barrel. **d,** TamAB hybrid barrel structure superimposition with BAM-EspP complex (7YE6) with twelve EspP beta-strands outside of the BamA barrel. **e,** TamAB hybrid barrel structure superimposition with BAM-EspP complex (8BNZ) with twelve EspP beta-strands outside of the BamA barrel. **f,** TamAB hybrid barrel structure superimposition with BAM-EspP complex (7TT0) with three EspP beta-strands outside of the BamA barrel. **g,** TamAB hybrid barrel structure superimposition with BAM-EspP complex (8BO2) with twelve EspP beta-strands outside of the BamA barrel, and EspP near maturation. **h,** TamAB hybrid barrel structure superimposition with BAM-EspP complex (6V05) with fourteen BamA beta-strands outside of the BamA barrel.

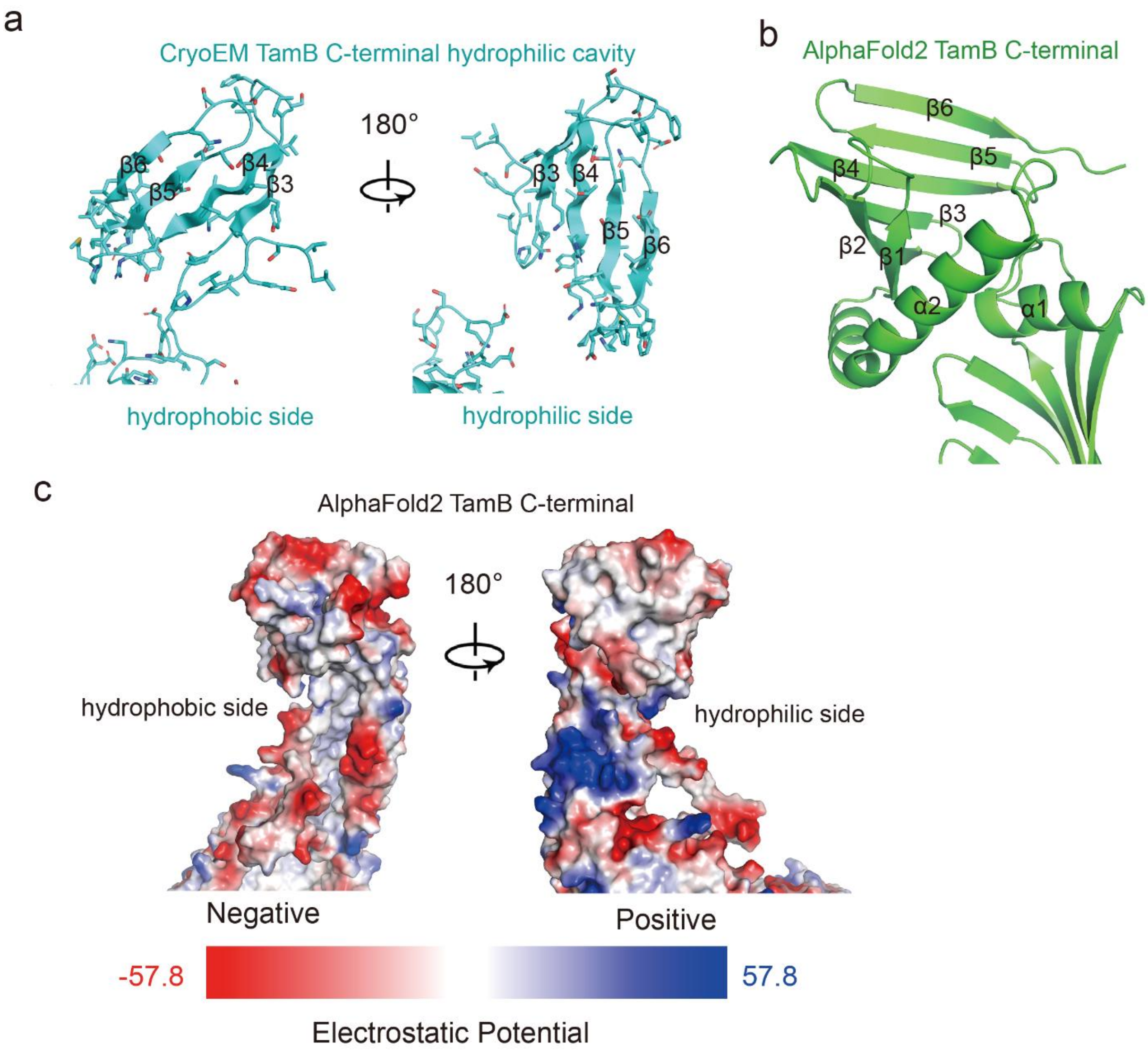

Supplementary Figure. 5. | The beta sheet of the C-terminal domain of TamB is amphipathic. a, The structure of the C-terminal beta sheet of the TamB cryo-EM structure, showing the hydrophobic and hydrophilic sides. **b,** The C-terminal six beta-stranded sheet with alpha1 and alpha2 structure was predicted by AlphaFold2. **c,** Electrostatic potential maps of the TamB structure predicted by AlphaFold2. The outside surface is hydrophilic and the cavity is hydrophobic.

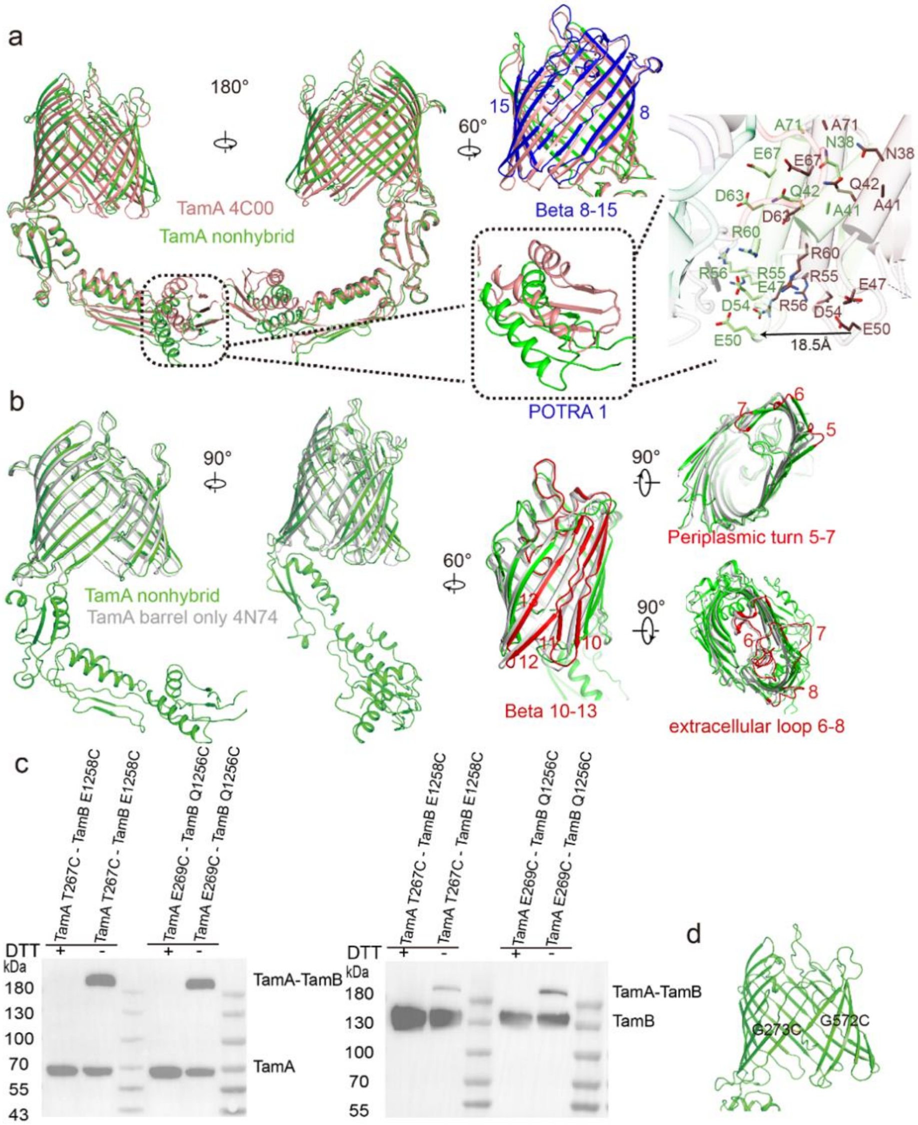

Supplementary Figure. 6. | TamA from the non-hybrid barrel structures superimposition with crystal structures of whole length and barrel only TamA. a, TamAB non-hybrid barrel structure superimposition with the whole length TamA crystal structure (PDB code: 4C00), showing their beta strands 8-15 and POTRA1 conformational changes. **b,** TamAB non-hybrid barrel structure superimposition with the barrel only TamA crystal structure (PDB code: 4N74), showing their beta strands 10-13, PT5-7 and ECL6-8 conformational changes. **c,** Disulfide bond formation between TamA β1 and TamB β6 confirmed by western blotting. All experiments were repeated for three times. **d,** TamA β1 residue Gly273 and β16 Gly572 double cysteine mutation could form a disulfide bond.

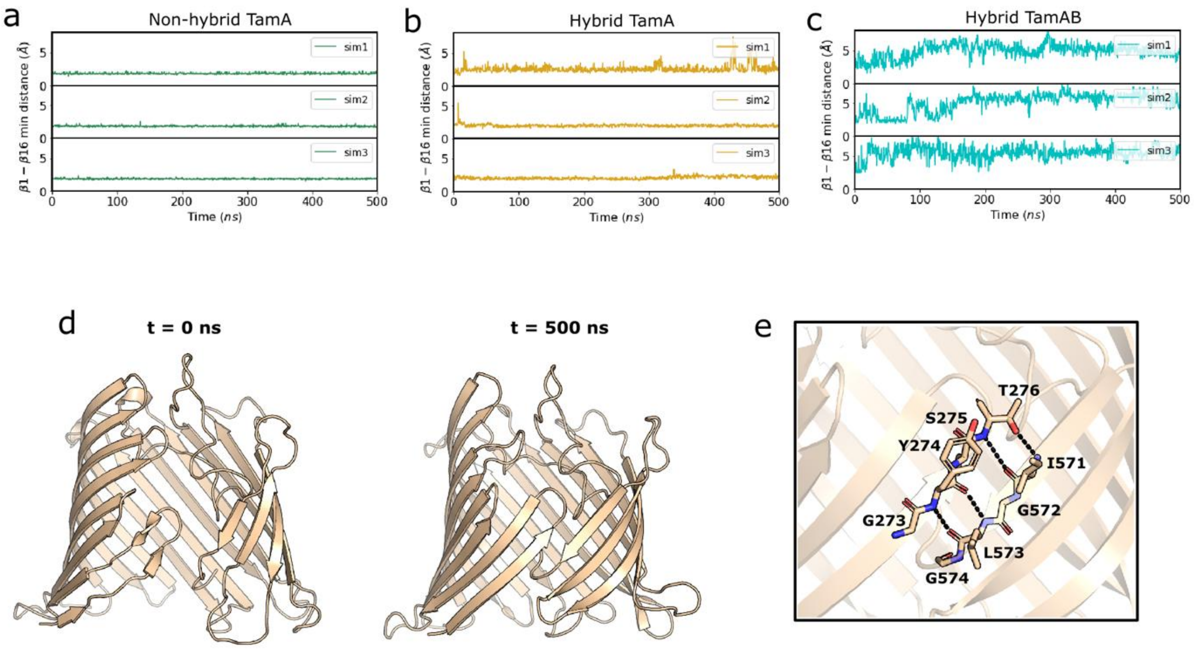

Supplementary Figure. 7. | Molecular dynamics simulations of TamA barrel. Minimum distance between TamA β1 and β16 strands for the **a,** Non-hybrid TamA barrel, **b,** Hybrid TamA barrel and **c,** hybrid TamAB barrel. **d,** Initial and final frames of hybrid TamA barrel without TamB strands. **e,** Hydrogen bonds between TamA β1 and β16 strands. After removing TamB strands from the hybrid TamAB barrel, TamA adopts the closed conformations and hydrogen bonds between TamA β1 and β16 strands are restored.

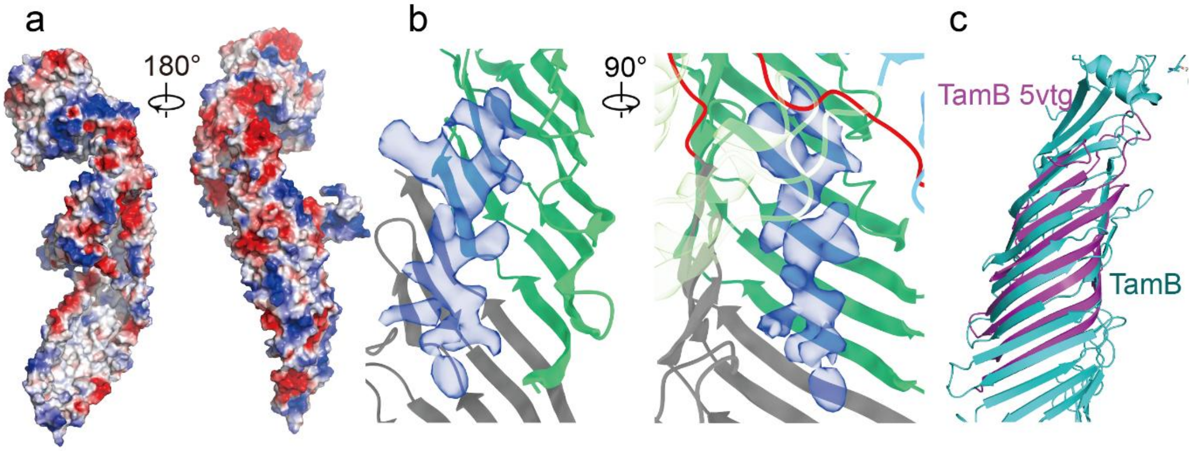

Supplementary Figure. 8. | Cryo-EM density of the TamB periplasmic domain suggests that TamB binds phospholipid. a, Electrostatic potential map of TamAB. TamB folds a hydrophobic cavity. **b,** Density in the TamB periplasmic domain is potentially Amphipol 8-35, **c,** Crystal structure of TamB (PDB code: 5VTG) superimposes well with the part of the TamB periplasmic cryo-EM structure.

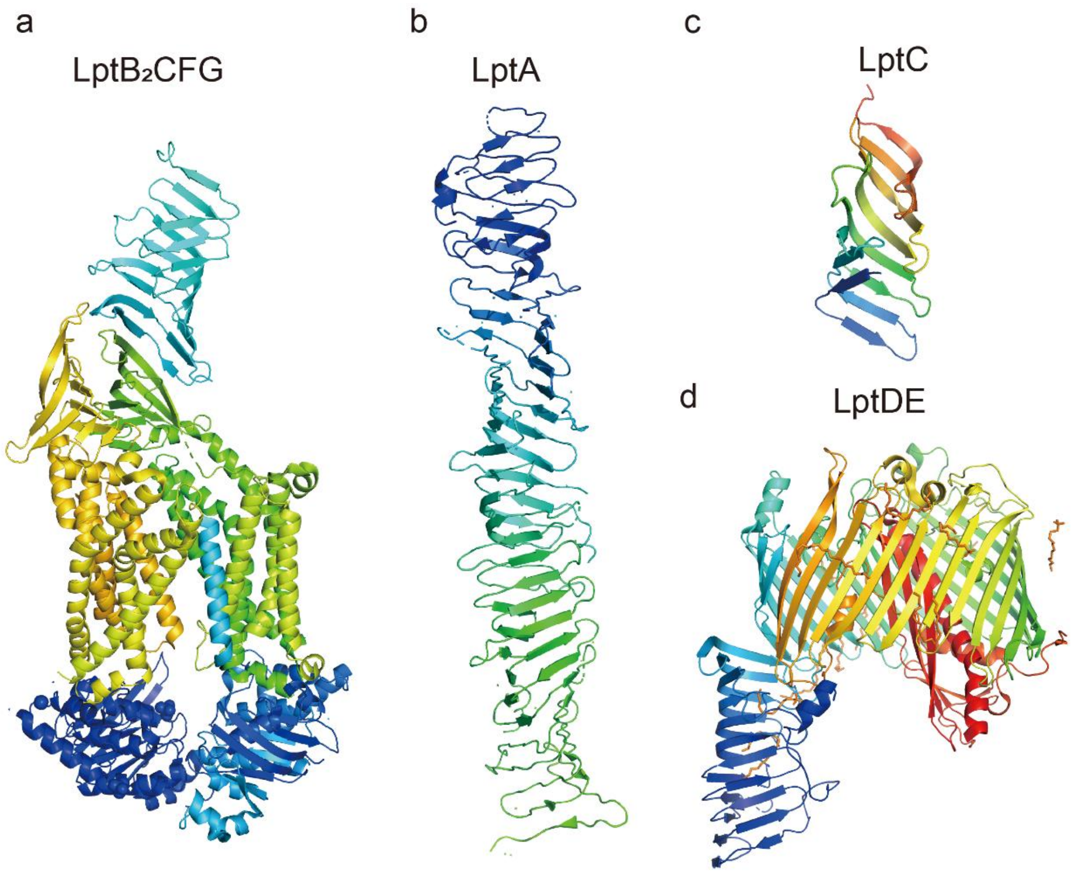

Supplementary Figure. 9. | Lipopolysaccharide transport proteins share similar fold with TamB. a, $LptB_2CFG$ structure (PDB code: 6MIT). **b,** Crystal structure of LptA (PDB code: 2R1A). **c,** Crystal structrure of LptC (PDB code: 3MY2). **d,** Crystal structure LptDE (PDB code: 4Q35).

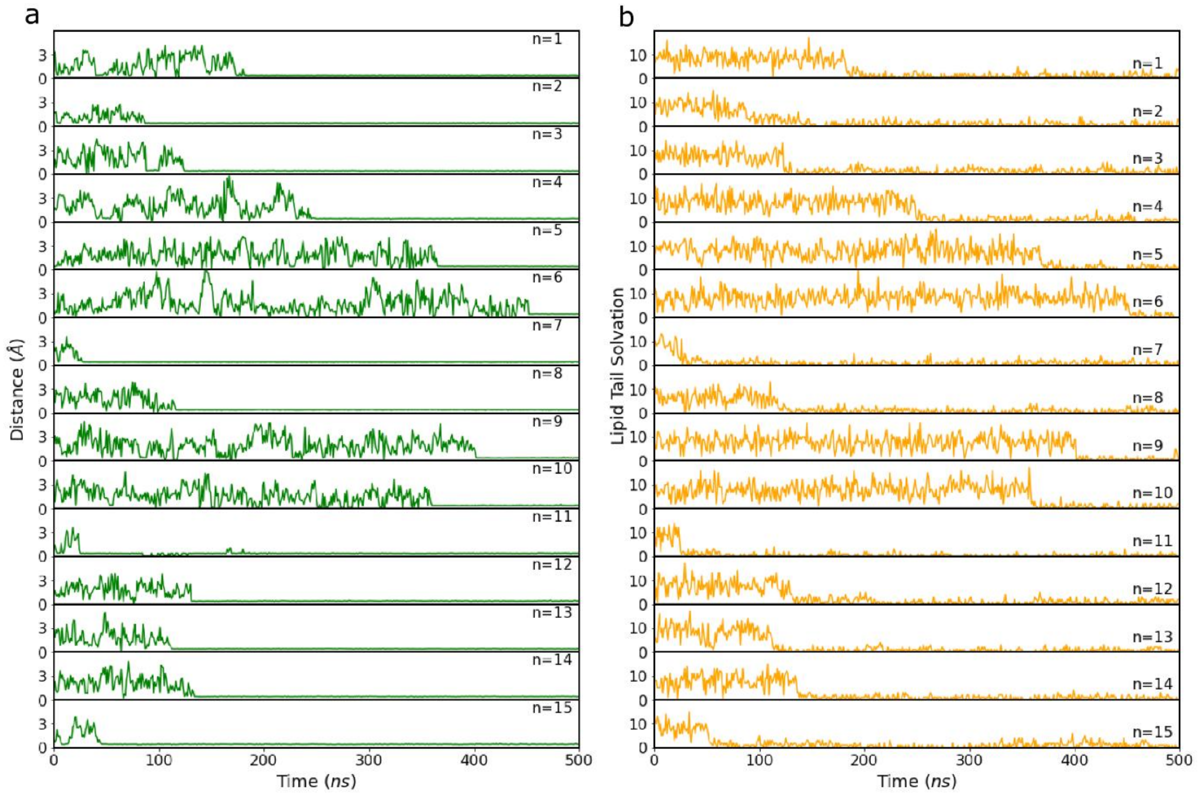

Supplementary Figure. 10. | Lipid binding to TamB. Time trace of **a,** The minimum distance between the lipids and TamB and **b,** The lipid tail solvation for each lipid addition step.

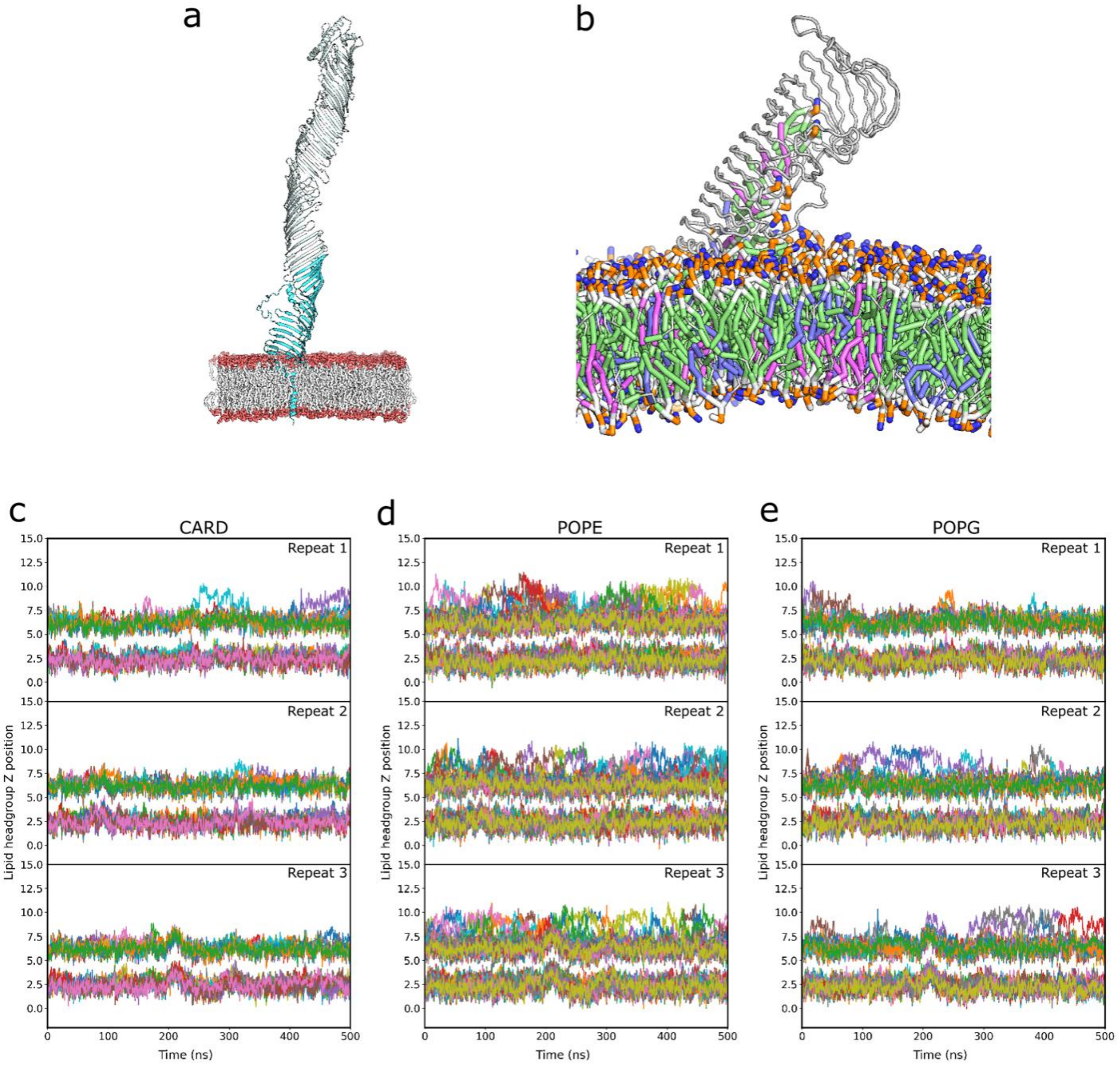

Supplementary Figure. 11. | Lipid binding to TamB. a, Full length model of TamB embedded in a POPE:POPG:CARD lipid bilayer, with the N-terminal segment considered in our CG MD simulations. **b,** Representative frame from coarse-grained simulations showing lipids of different chemical nature occupying the hydrophobic cavity of TamB. POPE molecules are shown in green, POPG in blue, and cardiolipin in magenta. Time trace of the lipid headgroup z positions for **c,** cardiolipin, **d,** POPE and **e,** POPG. Each line indicates the z position of one lipid headgroup.

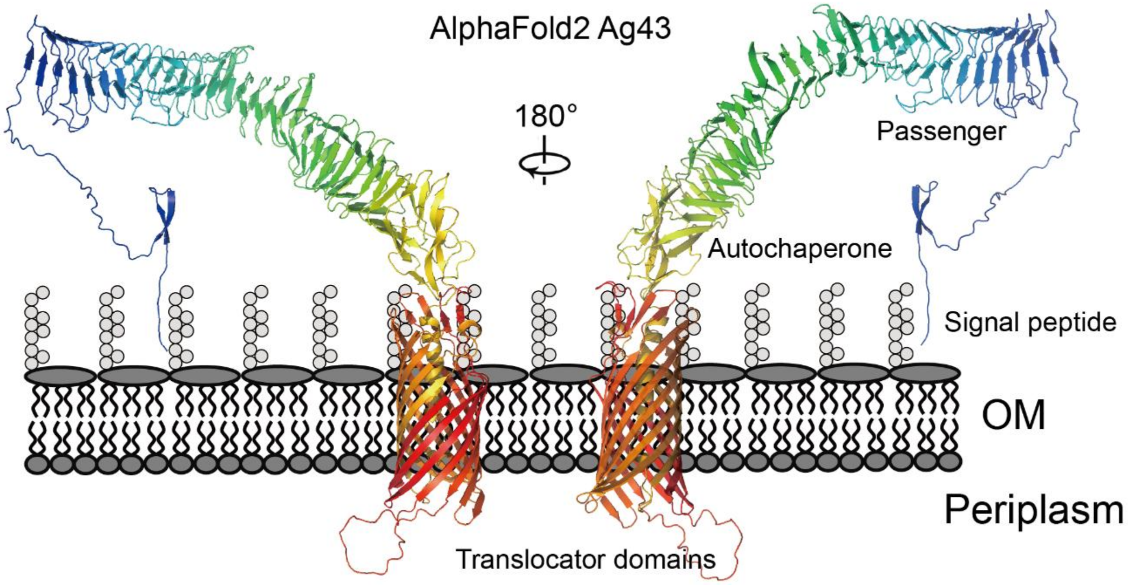

Supplementary Figure. 12. | The Ag43 structure was predicted by AlphFold2. The Ag43 is reported to be folded in the OM by TamAB, while the alpha -domain is exported to the outer surface of the bacterium, where it is involving in bacterial aggregation and biofilm formation.

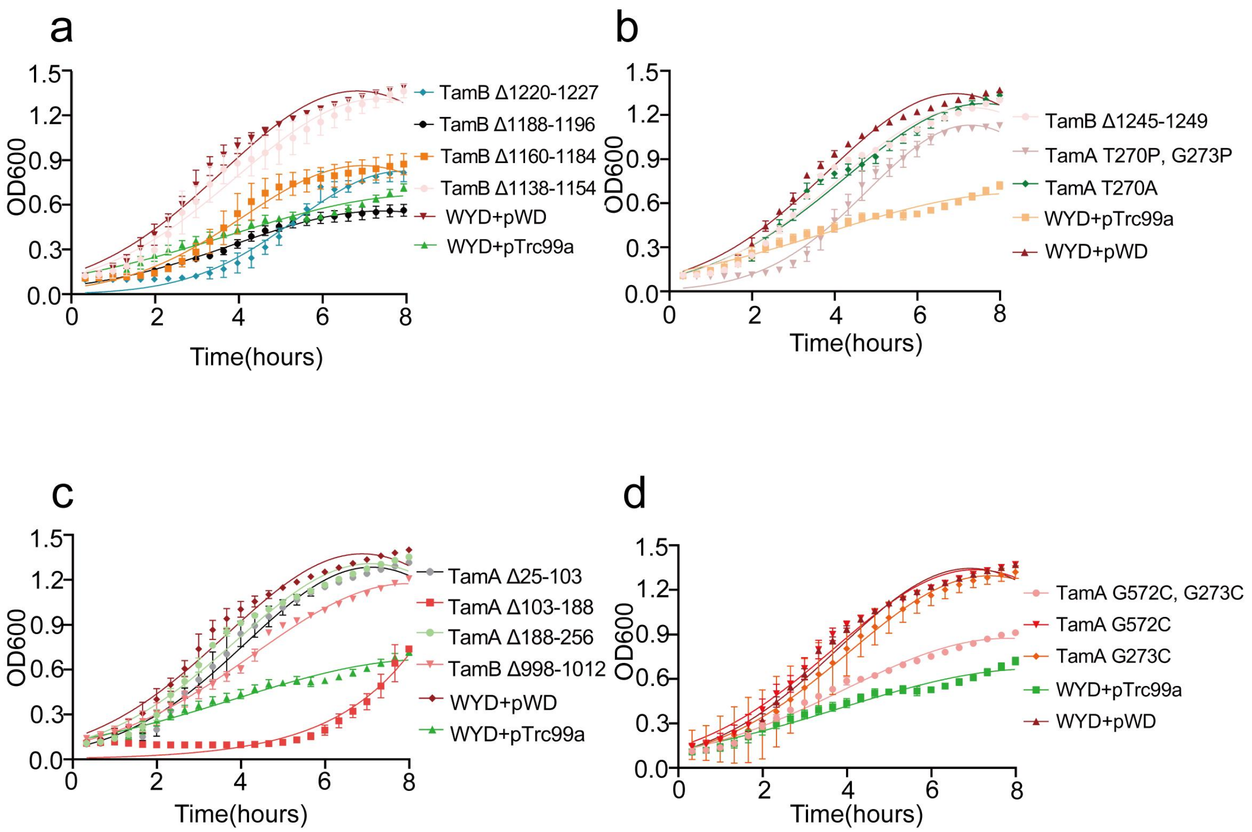

Supplementary Figure. 13. | The bacterial growth curve of the mutants and wild types. **a-d**, Growth curves were performed at 37℃ and measured by monitoring the OD600. All experiments were repeated for three times. Data are presented as mean ± SD.

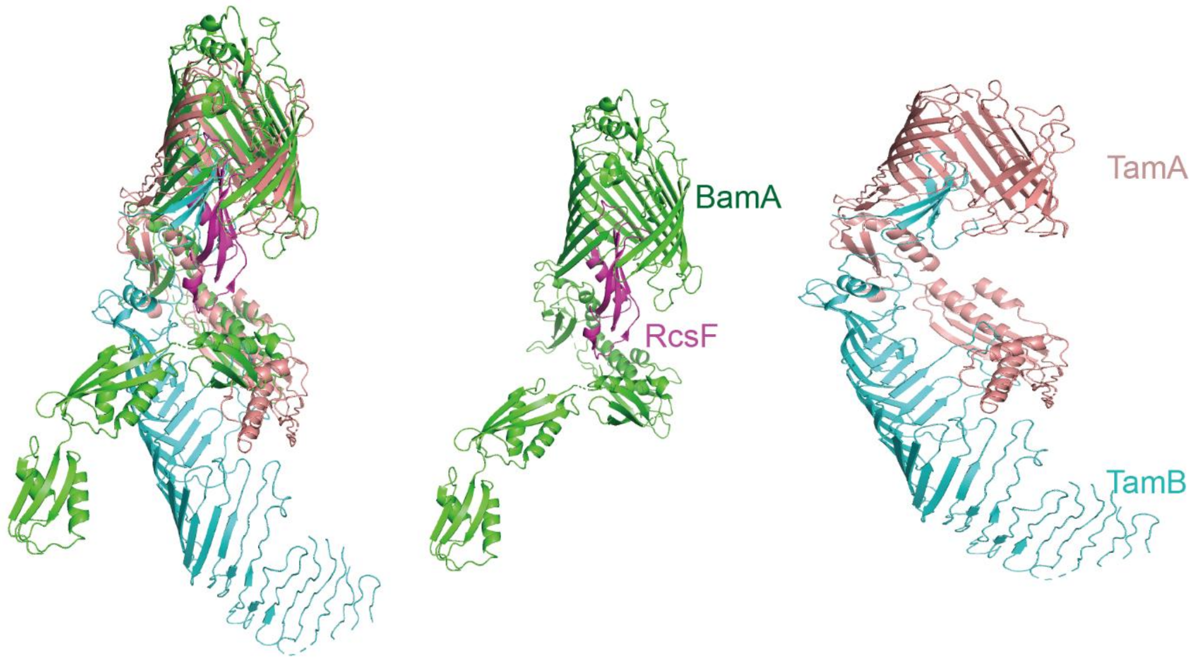

Supplementary Figure. 14. | TamAB hybrid barrel structure superimposition with with the BamA:RcsF structure (PDB code 6T1W), which showed that the TamA and BamA have quite different structures. TamB last beta strand (β6) interacts with TamA barrel β1 and the TamB C-terminal domain β3-6 fold into the the lumen of the TamA barrel, while half of RcsF is inserted in the barrel of the BamA with different interaction module

Supplementary Table. 15. | Cryo-EM structure determination parameters and model statistics

	Hybrid TamAB<br>PDBID: 9XDC<br>EMDBID: EMD-66762	Non-hybrid TamAB<br>PDBID: 9XDD<br>EMDBID: EMD-66763
**Data collection and processing**		
Microscope	Titan krios	Titan krios
detector	K3	K3
Magnification	105000	105000
Voltage (KV)	300	300
Electron exposure (e/Å2)	50	50
Defocus range (um)	-1 to -3	-1 to -3
Pixel size (Å)	0.84	0.84
Symmetry imposed	C1	C1
Initial particle images (no.)	25,588,098	25,588,098
Final particle images (no.)	110,602	110,592
Map resolution (Å)	3.69 Å	3.82 Å
FSC threshold	0.143	0.143
**Refinement**		
Map sharpening B factor (Å)	-113.8	-118.7
Model composition		
Non-hydrogen atoms	6763	6458
Protein residues	888	841
Ligands	0	0
B-factors(Å)		
Protein	50.17	59.70
R.M.S deviations		
Bond length (Å)	0.002	0.003
Bond angles ()	0.494	0.545
Validation		

Molprobity score	1.83	1.92
Clash score	6.63	9.21
Ramachandran plot		
Favored (%)	92.50	93.41
Allowed (%)	7.27	6.47
Outliers (%)	0.23	0.12

Supplementary Table. 16. | Bacterial strains and plasmids used in this study

Strain	genotype	source
BL21 (DE3)	F^{-} *ompT hsdS*$_B$ *(r*$_B^-$ *m*$_B^-$*) gal dcm (DE3)*	CAT#: EC1002
C43 (DE3)	F^{-}*ompT hsdS*$_B$ *(r*$_B^-$ *m*$_B^-$*) gal dcm (DE3)*	CAT#: EC1040
WDY	*ΔtamAB ΔydbH araC–arcBAD-yhdP*	MG1655
MG1655Δ*tamAB*	*tamAB* deletion	MG1655
Mg1655-pBad-BamA	the *araC–arcBAD*-controlled BamA strain	MG1655
MG1655	F^{-} *lambda*$^-$ *ilvG*$^-$ *rfb*$^-$ *50 rph-1*	CAT#: DL2030
**Plasmid**	**characteristic**	**source**
pTrc99a-*tamAB* (8×His)	*tamAB* gene in pTrc99a	This study
pWD	*tamA* (Flag) *tamB* (Myc) in pTrc99a	This study
pTrc99a-SUMO-*tamB*$_{43\text{-}1156}$	N-terminal SUMO tag fusion *tamB*$_{43\text{-}1156}$ in pTrc99a	This study
pBAD33-*ag43*	*ag43* gene with Flag tag after residue 58 and Myc tag after residue 558 in pBAD33	This study
pBAD33-*ag43*$_{\Delta 755\text{-}771}$	755-771 deleted Ag43 in pBAD33-*ag43*	This study
pTrc99a-*tamA* (T270P, G273P) *tamB*	T270P and G273P double mutations on TamA in pWD	This study
pTrc99a-*tamA* (T270A)	T270A single mutation on	This study

tamB	TamA in pWD	
pTrc99a-*tamAtamB* (Δ1245-1249)	1245-1249 deleted TamB in pWD	This study
pTrc99a-*tamAtamB* (Δ1220-1227)	1220-1227deleted TamB in pWD	This study
pTrc99a-*tamAtamB* (Δ1188-1196)	1188-1196 deleted TamB in pWD	This study
pTrc99a-*tamAtamB* (Δ1160-1184)	1160-1184 deleted TamB in pWD	This study
pTrc99a-*tamAtamB* (Δ1137-1154)	1137-1154deleted TamB in pWD	This study
pTrc99a-*tamA* (V548D) *tamB*	V548D single mutation on TamA in pWD	This study
pTrc99a-*tamA* (G572C, G273C) *tamB*	G572C and G273C double mutations on TamA in pWD	This study
pTrc99a-*tamA* (G572C) *tamB*	G572C single mutation on TamA in pWD	This study
pTrc99a-*tamA* (G273C) *tamB*	G273C single mutation on TamA in pWD	This study
pTrc99a-*tamA* (Δ25-103) *tamB*	25-103 deleted TamA in pWD	This study
pTrc99a-*tamA* (Δ103-188) *tamB*	103-188 deleted TamA in pWD	This study
pTrc99a-*tamA* (Δ188-263) *tamB*	188-263 deleted TamA in pWD	This study
pTrc99a-*tamAtamB* (Δ998-1012)	998-1012 deleted TamB in pWD	This study
pTrc99a-*tamA* (T267C) *tamB*	T267C single mutation on TamA in pWD	This study

pTrc99a-*tamA* (E269C) *tamB*	E269C single mutation on TamA in pWD	This study
pTrc99a-*tamA* (T267C) *tamB* (E1258C)	T267C and E1258C double mutations on TamAB in pWD	This study
pTrc99a-*tamA* (E269C) *tamB* (Q1256C)	E269C and Q1256C double mutations on TamAB in pWD	This study
pTrc99a-*tamA* (G271C) *tamB* (L1254C)	G271C and L1254C double mutations on TamAB in pWD	This study
pTrc99a-*bamABCDE*	*bamA*(Flag)*BCDE* in pTrc99a	This study
pTrc99a-*bamA* (G431C)	G431C single mutation on BamA in pTrc99a-*bamABCDE*	This study
pBAD33-$ag43_{\Delta 755\text{-}771}$ (Q1033C)	Q1033C single mutation on Ag43 in pBAD33-$ag43_{\Delta 755\text{-}771}$	This study
pBAD33-$ag43_{\Delta 755\text{-}771}$ (N1037C)	N1037C single mutation on Ag43 in pBAD33-$ag43_{\Delta 755\text{-}771}$	This study
pBAD33-$ag43_{\Delta 755\text{-}771}$ (T1039C)	T1039C single mutation on Ag43 in pBAD33-$ag43_{\Delta 755\text{-}771}$	This study
pTrc99a	empty vector	Addgene

Supplementary Table. 17. | Coarse-Grained Binding simulations setup

Coarse-Grained Binding Simulations		
**System**	**# POPE**	**# Water**
Step 1	1	15184
Step 2	2	9676
Step 3	3	14090
Step 4	4	14593
Step 5	5	10588
Step 6	6	9491
Step 7	7	13737
Step 8	8	10974
Step 9	9	13426
Step 10	10	12118
Step 11	11	11263
Step 12	12	12040
Step 13	13	10990
Step 14	14	13209
Step 15	15	14412

Supplementary Table. 18. | All-atom MD simulations setup

All-Atom MD Simulations				
**System**	**# POPE**	**# POPG**	**# REMP**	**# Water**
Hybrid TamA	277	69	140	77096
Non-Hybrid TamA	235	58	130	65492
Hybrid TamAB	294	73	153	82356
