# Supplementary material for: Structural basis of outer membrane biogenesis by the TamAB translocase
Source: Nat Commun. 2026 Jan 13;17:437. doi: 10.1038/s41467-025-67115-7 (PMC12800296; doi:10.1038/s41467-025-67115-7)
Supplement: Supplementary file 2 — Reporting Summary [file 41467_2025_67115_MOESM2_ESM.pdf]

nature portfolio

Corresponding author(s): ChangJiang Dong; Zhengyu Zhang

Last updated by author(s): Nov 18, 2025

Reporting Summary

Nature Portfolio wishes to improve the reproducibility of the work that we publish. This form provides structure for consistency and transparency in reporting. For further information on Nature Portfolio policies, see our Editorial Policies and the Editorial Policy Checklist.

Statistics

For all statistical analyses, confirm that the following items are present in the figure legend, table legend, main text, or Methods section.

n/a	Confirmed	
☐	☒	The exact sample size (*n*) for each experimental group/condition, given as a discrete number and unit of measurement
☐	☒	A statement on whether measurements were taken from distinct samples or whether the same sample was measured repeatedly
☒	☐	The statistical test(s) used AND whether they are one- or two-sided<br>*Only common tests should be described solely by name; describe more complex techniques in the Methods section.*
☒	☐	A description of all covariates tested
☒	☐	A description of any assumptions or corrections, such as tests of normality and adjustment for multiple comparisons
☐	☒	A full description of the statistical parameters including central tendency (e.g. means) or other basic estimates (e.g. regression coefficient) AND variation (e.g. standard deviation) or associated estimates of uncertainty (e.g. confidence intervals)
☒	☐	For null hypothesis testing, the test statistic (e.g. *F*, *t*, *r*) with confidence intervals, effect sizes, degrees of freedom and *P* value noted<br>*Give P values as exact values whenever suitable.*
☒	☐	For Bayesian analysis, information on the choice of priors and Markov chain Monte Carlo settings
☒	☐	For hierarchical and complex designs, identification of the appropriate level for tests and full reporting of outcomes
☒	☐	Estimates of effect sizes (e.g. Cohen's *d*, Pearson's *r*), indicating how they were calculated

Our web collection on statistics for biologists contains articles on many of the points above.

Software and code

Policy information about availability of computer code

Data collection	CyroSPARC
Data analysis	CyroSPARC, ChimeraX, Phenix, Pymol,Coot

For manuscripts utilizing custom algorithms or software that are central to the research but not yet described in published literature, software must be made available to editors and reviewers. We strongly encourage code deposition in a community repository (e.g. GitHub). See the Nature Portfolio guidelines for submitting code & software for further information.

Data

Policy information about availability of data

All manuscripts must include a data availability statement. This statement should provide the following information, where applicable:

- Accession codes, unique identifiers, or web links for publicly available datasets
- A description of any restrictions on data availability
- For clinical datasets or third party data, please ensure that the statement adheres to our policy

The density maps of cryo-EM and coordinates for TamAB machinery in hybrid (open) barrel and non-hybrid (semi-closed) states were deposited in the Protein Data Bank at the access codes EMD-60411 and 8ZSC, EMD-60412 and 8ZSD, respectively.

Research involving human participants, their data, or biological material

Policy information about studies with human participants or human data. See also policy information about sex, gender (identity/presentation), and sexual orientation and race, ethnicity and racism.

Reporting on sex and gender	Not involved
Reporting on race, ethnicity, or other socially relevant groupings	Not involved
Population characteristics	Not involved
Recruitment	Not involved
Ethics oversight	Not involved

Note that full information on the approval of the study protocol must also be provided in the manuscript.

Field-specific reporting

Please select the one below that is the best fit for your research. If you are not sure, read the appropriate sections before making your selection.

- [x] Life sciences
- [ ] Behavioural & social sciences
- [ ] Ecological, evolutionary & environmental sciences

For a reference copy of the document with all sections, see nature.com/documents/nr-reporting-summary-flat.pdf

Life sciences study design

All studies must disclose on these points even when the disclosure is negative.

Sample size	Cryogenic electron microscopy data was collected at Wuhan University. The datasets were collected in this study.
Data exclusions	Not involved
Replication	All experiments were repeated for at least three times.
Randomization	Not involved
Blinding	Not involved

Reporting for specific materials, systems and methods

We require information from authors about some types of materials, experimental systems and methods used in many studies. Here, indicate whether each material, system or method listed is relevant to your study. If you are not sure if a list item applies to your research, read the appropriate section before selecting a response.

Materials & experimental systems

n/a	Involved in the study	
[ ]	[x]	Antibodies
[x]	[ ]	Eukaryotic cell lines
[x]	[ ]	Palaeontology and archaeology
[x]	[ ]	Animals and other organisms
[x]	[ ]	Clinical data
[x]	[ ]	Dual use research of concern
[x]	[ ]	Plants

Methods

n/a	Involved in the study	
[x]	[ ]	ChIP-seq
[x]	[ ]	Flow cytometry
[x]	[ ]	MRI-based neuroimaging

Antibodies

Antibodies used	anti-Flag (1:1000 dilution) (Sigma, Catalogue No: F1804) , anti-Myc monoclonal antibody (1:5000 dilution) (Sigma, Catalogue No: 4439), goat anti-mouse IgG antibody(proteintech, Catalogue No: SA00001-1),, 6*His, His-Tag Monoclonal antibody(1:5000 dilution) (Proteintech,Catalogue No. 66005-1-Ig),AF488-labeled Goat Anti-Mouse IgG (H+L)(Beyotime Biotechnology, A0428)
Validation	The application and specificity of all the antibodies are validated by the companies.

Plants

Seed stocks	Not involved
Novel plant genotypes	Not involved
Authentication	Not involved
